# Time to get deep

**DOI:** 10.7554/eLife.100755

**Published:** 2024-07-17

**Authors:** Max Schulz, Malte Wöstmann

**Affiliations:** 1 Department of Psychology, University of Lübeck Lübeck Germany

**Keywords:** spatial attention, subcortical structures, oscillations, alpha, hemispheric asymmetry, Human

## Abstract

Asymmetries in the size of structures deep below the cortex explain how alpha oscillations in the brain respond to shifts in attention.

**Related research article** Ghafari T, Mazzetti C, Garner K, Gutteling T, Jensen O. 2024. Modulation of alpha oscillations by attention is predicted by hemispheric asymmetry of subcortical regions. *eLife* **12**:RP91650. doi: 10.7554/eLife.91650.

As you read this text, you are probably focusing on a screen or piece of paper directly in front of you. This ability to focus on a specific object relies on the brain filtering out visual distractions from the surrounding area (Desimone and Duncan, 1995). However, if you wanted, you could suddenly shift your attention to the left or right, without moving your eyes. Understanding more about the relationship between attention and the anatomy of the brain is fundamental for research in neuroscience.

Previous studies have shown that attention is controlled by the cortex, the outer layer of the brain, which is thought to implement cognition, language, reasoning and other higher-order brain functions. However, much less is known about how structures deep within the brain affect this process. This is partly because most of the techniques commonly used to image brain activity are good at measuring the activity of neurons in the cortex but less so in deeper subcortical structures. Now, in eLife, Ole Jensen and colleagues at the University of Birmingham, University of New South Wales, and CERMEP-Imagerie du Vivant – including Tara Ghafari as first author – report how they used two brain imaging techniques to investigate the role of subcortical structures in spatial attention (Ghafari et al., 2024).

First, the team used a technique called magnetoencephalography (MEG) to measure magnetic fields generated by the electrical activity of neurons in the cortical layer. This method was applied to the brains of 33 individuals as they performed a task that required them to shift their attention between faces on the left and right of a computer screen (Gutteling et al., 2022). The same group of people were also placed in a magnetic resonance imaging (MRI) scanner to assess the size of two brain structures, the thalamus and basal ganglia, both of which sit beneath the cortex.

When our attention shifts to the left or right, the balance of waves in the left versus the right hemisphere of the brain changes. This is particularly true for a pattern of electrical activity known as the alpha wave which repeats roughly ten times per second (first discovered by Berger, 1929). It is believed that this asymmetry reflects one side of the brain focusing on the relevant input, while the other suppresses distractions from the surrounding environment (Schneider et al., 2022).

However, the human brain is not perfectly symmetrical. For instance, structures which are present on both sides of the brain (such as the thalamus) might be larger in the right hemisphere in some individuals, but larger in the left in others. On top of this, the response of alpha waves to attention is also asymmetrical: a shift in attention might lead to a larger change in the alpha waves in one hemisphere for some individuals, and the opposite hemisphere for others (Mazzetti et al., 2019).

Ghafari et al. set out to find whether the size of subcortical structures in the left and right hemispheres correlates with the asymmetry in alpha modulations. They found that whichever brain hemisphere had the larger caudate nucleus or globus pallidus (two structures that make up the basal ganglia) also displayed a higher level of alpha wave modulation. However, this effect was reversed for the thalamus, with higher levels of modulation happening in the hemisphere with the smaller thalamus (Figure 1). Moreover, Ghafari et al. also report what happens to these relationships when various features of the relevant stimuli and irrelevant distractors used in the experiments are changed.

**Figure 1.**
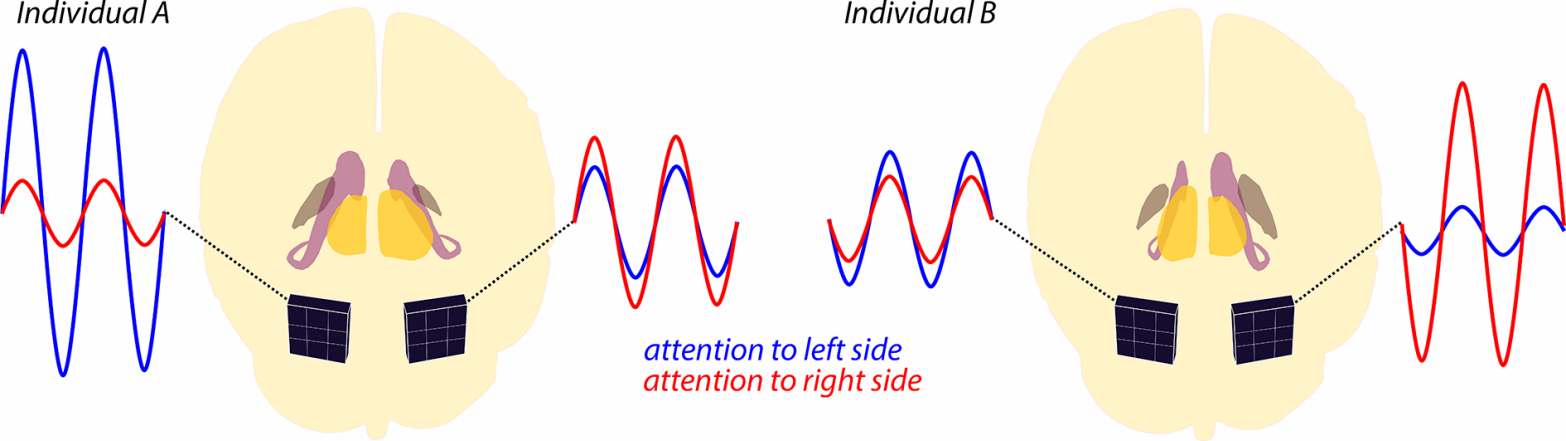
Alpha wave modulation during a spatial attention task. The brains of two individuals are shown schematically from the back, with subcortical structures (thalamus, caudate nucleus and globus pallidus) highlighted in different colors. Note that for individual A, the thalamus (yellow) in the left hemisphere is slightly smaller than the thalamus in the right hemisphere. There are also asymmetries in the sizes of the caudate nucleus (purple) and globus pallidus (brown), and also for all three structures in individual B. MEG sensors (black squares) attached to the posterior cortex record alpha waves as the individuals shift their attention to the left or right side of their visual field. For both individuals, shifting attention to the left leads to increased alpha waves in the left hemisphere (blue lines), and shifting attention to the right leads to increased alpha waves in the right hemisphere (red lines). The extent of this modulation of the alpha waves is related to the size of various subcortical structures: a larger thalamus on one side of the brain – the right hemisphere of individual A, the left hemisphere of individual B – correlates with a stronger alpha modulation in the opposite hemisphere. Conversely, a larger caudate nucleus or globus pallidus correlates with stronger alpha modulation in the same hemisphere.

Image Credit: Shapes of the brain and subcortical structures adapted from an interactive brain model powered by the Wellcome Trust and developed by Matt Wimsatt and Jack Simpson, Society for Neuroscience (2017).

These new findings will help us understand the underlying subcortical circuitry that controls how spatial attention is allocated in humans. Not least, this work paves the way for further research on how changes in the subcortical regions in neurological disorders such as Alzheimer’s disease or dementia alter the behavior of brain waves in the cortex.

Since the results of the present research are purely correlational, it remains unclear whether the asymmetry of subcortical structures is responsible for the asymmetry in alpha wave modulation. Furthermore, attention is just one of many perceptual and cognitive processes that modulate alpha waves (Clayton et al., 2018). It will thus be important for future studies to test if the results are specific to spatial attention or apply more generally to other cognitive processes and to other types of brain waves.
